# Migration of wheat stripe rust from the primary oversummering region to neighboring regions in China

**DOI:** 10.1038/s42003-025-07789-3

**Published:** 2025-03-03

**Authors:** Yuxiang Li, Siyue Zhang, Di Liu, Taixue Zhang, Zhibo Zhang, Jingchen Zhao, Bo Zhang, Shiqin Cao, Xiangming Xu, Qiang Yao, Xiaoping Hu

**Affiliations:** 1 State Key Laboratory for Crop Stress Resistance and High-Efficiency Production, Key Laboratory of Plant Protection Resources and Pest Integrated Management of Ministry of Education, Key Laboratory of Integrated Pest Management on Crops in Northwestern Loess Plateau of Ministry of Agriculture and Rural Affairs, and College of Plant Protection, Northwest A&F University, Taicheng Road 3, Yangling, Shaanxi China; 2 Institute of Plant Protection, Gansu Academy of Agricultural Sciences, Lanzhou, China; 3 Pest & Pathogen Ecology, NIAB East Malling, West Malling, Kent, UK; 4 Qinghai Provincial Key Laboratory of Agricultural Integrated Pest Management Scientific Observing and Experimental Station of Crop Pest in Xining, Ministry of Agriculture and Rural Affairs, Academy of Agriculture and Forestry Science, Qinghai University, Xining, Qinghai China

**Keywords:** Fungal genetics, Fungal biology

## Abstract

Changing climate and changes in cropping systems have greatly affected outbreaks of plant diseases. Wheat stripe rust is a disease posing a threat to global wheat production, caused by *Puccinia striiformis* f. sp. *tritici* (*Pst*). *Pst* oversummering regions play a crucial role in the emergence of new races in China. To unveil the migration pattern of oversummering to adjacent regions, we develop a set of KASP-SNP marker from 28 *Pst* whole-genome sequences to investigate the population structure in the oversummering and its adjacent regions. A set of 19 Chinese wheat differentials is used to characterize the virulence patterns of 308 sampled *Pst* isolates. By integrating virulence characterization, population genetic analysis, air trajectory simulation and field disease monitoring, two main *Pst* dispersal routes are identified. Inocula from Eastern Qinghai are dispersed to Western and Eastern Liupan Mountain, and reach Guanzhong Plain. The second route originates from Middle Gansu, then through Longnan, and reaches the Guanzhong Plain via Eastern Liupan Mountain. Both dispersal routes result in *Pst* inoculum spreading to the Huang-Huai-Hai region, the main wheat-growing region in China. The proposed migration routes can be used to develop disease management strategies at a regional and national scale.

## Abstract (web summary)

Migration patterns of the wheat stripe rust pathogen from oversummering regions to adjacent areas were identified, revealing two dispersal routes of disease spread and providing insights for regional and national disease management strategies.

## Introduction

The increasing prevalence of plant disease outbreaks poses a substantial threat to global food safety^1,2^. Long-distance dispersal of plant pathogens facilitates their invasion of new territories, ultimately contributing to large-scale disease epidemics^3,4^. Diseases caused by airborne pathogens, such as cereal rust, blight, and powdery mildew, are characterized by inoculum dispersal over extended distances up to thousands of kilometers^5–7^. Understanding the spatial epidemic patterns and pathogen population biology is needed for formulating effective management strategies at a range of spatial scales.

Wheat stripe rust, caused by *Puccinia striiformis* f. sp. *tritici* (*Pst*), is a threat to global food security, impacting more than 60 countries^8,9^. *Pst* is a macrocyclic and heteroecious fungus, which requires five different spore stages and two unrelated hosts to complete its life cycle^8,10^. Although *Berberis* spp. was identified as an alternative host for *Pst* (leading to *Pst* sexual reproduction), *Pst* epidemics mainly rely on asexual urediniospores^11–13^. Regions where *Pst* can survive both winter and summer tend to have more frequent and severe epidemics than other regions, and these oversummering and overwintering regions are the sources of *Pst* inocula for other regions^3,14,15^. For instance, the Pacific Coast region in the USA, where *Pst* can both oversummer and overwinter, functions as the primary inoculum source for *Pst* on wheat crops grown in neighboring areas^8,16^. In China, *Pst* can occur all year-round in the northwest and southwest, significantly impacting the main wheat production region in the east^15,17,18^. Regions with both spring and winter wheat crops are likely to experience more severe epidemics than those regions with either spring or winter grown in the same season^8,10,19^.

Because of complicated topographies, diverse microclimates, and varying cropping systems in China, *Pst* exhibits a distinctive epidemic characteristic compared to other countries. Interregional *Pst* outbreaks occur nearly every year in China, resulting in an annual average yield loss of approximately 1 million tons^20–22^. Chinese National Wheat Rust Collaborative Group (CNWRCG) has been studying the epidemiology and management of *Pst* for more than 50 years. Based on field disease surveys and topographic features, wheat production areas within 97 to 135°E and 22 to 53°N was classified as the major *Pst* epidemic region^18,21^. Xinjiang and Xizang, separated by vast mountains and deserts with major epidemic regions, were considered as independent epidemic regions^23–25^. Fifteen epidemic regions were then proposed from the major epidemic region based on climatic characteristics and geographic features^18,21^. As *Pst* cannot oversummer in the majority of wheat-growing regions in China, *Pst* can only survive summer in those mountainous regions and plateaus with relatively high altitudes and low temperatures. Main *Pst* oversummering regions in China include the northwest (eastern Qinghai, southern Gansu, eastern Gansu and southern Ningxia), southwest (Yunnan, Guizhou, and northwest of Sichuan), the Himalayan region (Xizang), and western Xinjiang^15,18,19,21^. Given the topographic features and geographical distance of the Himalayan region and western Xinjiang from the main wheat-growing regions in the Huang-Huai-Hai plain, dispersal of *Pst* inocula from these two regions to the Huang-Huai-Hai plain is likely to be infrequent^23–25^. Although recent findings suggest that inocula in the southwest contributes to *Pst* development in the main wheat-growing area, *Pst* inoculum from the northwest oversummering region is still considered as the main infective source for the main wheat-growing areas^15,26–29^.

*Pst* population is most diverse in the northwest oversummering region in China^15,20,30,31^, which encompasses Western and Eastern Liupan Mountain, Southern Gansu, Middle Gansu, and Eastern Qinghai. Extensive research has been conducted to elucidate the relationship of *Pst* development in the oversummering region with its neighboring areas. Liang et al.^32^ found frequent gene flow of *Pst* between Gansu and Ningxia wheat-grown areas. *Pst* genetic variability in the subpopulation of Ningxia indicated that Liupan Mountain may have acted as an inoculum dispersal barrier affecting gene flows. Similar approaches were used to identify candidate *Pst* migration routes between Gansu and the Sichuan Basin, Xinjiang and Qinghai, and Gansu and Qinghai^33,34^. Liang et al.^31^ showed asymmetric *Pst* migrations between the northwest oversummering region and its neighboring areas, including southern Shaanxi and the Sichuan Basin. Li et al.^15^ proposed Longnan (southern Gansu), located in the northwest oversummering region, as the most significant contributor to *Pst* outbreaks in China. In addition, bi-directional migrations between Longnan and Sichuan Basin as well as Eastern Qinghai might also play important roles in *Pst* spreading to other regions. The one-directional migration from Longnan to Eastern Liupan Mountain was also proposed as another possible dispersal route^15,35^. Most of these studies focused on elucidating the relationship in *Pst* development between the oversummering region and its neighboring areas. However, given the large area of the oversummering region and the nature of geographic separation and variation in cropping systems within the region, insufficient attention has been given to understanding *Pst* dispersal routes among subpopulations within the northwest oversummering region.

The changing climate has led to significant changes in cropping systems in the *Pst* oversummering region. In Gansu, the wheat production area declined from 961,390 ha in 2005 to 711,270 ha in 2021, reducing the oversummering *Pst* inoculum in this region. Conversely, winter wheat production in the Eastern Qinghai increased from nearly zero to around 13,300 ha during the same period (https://data.stats.gov.cn/). Wheat cultivation in the Eastern Qinghai exhibits a staggered spatial pattern: both winter and spring wheat crops (total area exceeding 100,000 ha) are grown in locations with the altitude ranging from 1700 to 3200 meters above sea level. This pattern facilitates the spread of oversummering inocula from spring wheat to local winter wheat, significantly influencing *Pst* development in other areas.

To unravel the *Pst* migration pattern originating from oversummering regions under changing agriculture practices, the objectives of this study were to (i) characterize the virulence spectrum of *Pst* isolates sampled from main oversummering and adjacent regions, (ii) identify *Pst* genotypes using the newly developed KASP-SNP markers, (iii) propose *Pst* migration routes by integrating population genetic analyses, air trajectory simulations and field disease investigations. The findings of the present study will contribute to the formulation of regional and national disease management strategies.

## Results

### Virulence characterization of sampled *Pst* isolates

**Fig. 1:**
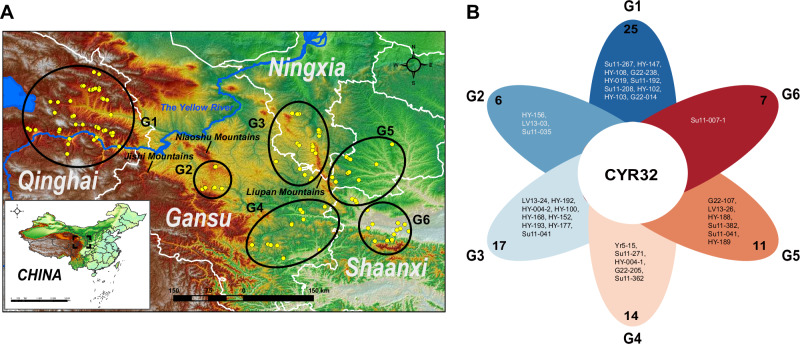
Geographic distribution and virulence characteristics of 308 *Puccinia striiformis* f. sp. *tritici* (*Pst*) isolates. **A** Six geographic regions in the oversummering and neighboring regions: G1 to G6, representing the Eastern Qinghai, Middle Gansu, Western Liupan Mountain, Longnan, Eastern Liupan Mountain, and Western Guanzhong Plain, respectively. Yellow dots indicate sampling sites. **B** Flower plot of *Pst* races identified across six geographic regions. Each petal corresponds to a different geographic region. The numbers on each petal indicate the number of races detected in that region, while the race names listed on the petal are exclusive to that region. CYR32 is placed in the center, highlighting that this race is detected in all geographic regions.

A total of 308 *Pst* isolates were sampled during 2021 and 2022 from the main *Pst* oversummering areas in Gansu, Qinghai, Ningxia provinces and the neighboring areas in Shaanxi province, covering 39 counties (Table S1). Based on geographic and topographic characteristics, these sampled sites were divided into six geographic populations, namely Eastern Qinghai (G1), Middle Gansu (G2), Western Liupan Mountain (G3), Longnan (G4), Eastern Liupan Mountain (G5), and Western Guanzhong Plain (G6) (Fig. 1a, Table S1).

Virulence pattern and race composition of all isolates were determined with selected 19 Chinese wheat differentials (WDs) containing various resistance genes. Out of the 19 WDs, more than 90% of isolates exhibited high virulence to Abbondanza (WD 5), Mentana (WD 4), Lovrin 10 (WD 16), Fengchan 3 (WD 11), and Suwon 11 (WD 14), indicating the ineffectiveness of *Yr9*, *Yr1*, and *YrSu* resistance genes (possibly other unknown resistance genes in these WDs). Zhong 4 (WD 4, containing unknown resistance gene(s)), *Triticum spelta* Album (WD 18, containing resistance gene *Yr5*), and Guinong 22 (WD 19, containing resistance genes *Yr10* and *Yr26*) appeared to be most resistant: only 13.96%, 0.32%, and 0.32% of 308 isolates were virulent against these three WDs, respectively (Table S2, Table S3).

A total of 150 *Pst* races were identified, comprising 51 named races and 99 new races. The 99 new *Pst* races consisted of 116 isolates; among these new races, 89 had only one single isolate (Table S3 and S4). CYR32 is most prevalent in all the races, accounting for 27.60%, followed by HY-008-1 (5.70%) and CYR34 (4.66%) (Table S3). Analyzing the distribution of the dominant race in different geographic populations revealed that CYR32 is the prevailing race in all populations except for Longnan (G4), where CYR34 holds dominance (Table S5) Among all the geographic populations, 25 races were identified in Eastern Qinghai (G1), which also harbors the highest number of exclusive races. In contrast, only 7 races were detected in Western Guanzhong Plain (G6), with just one exclusive race (Fig. 1b, Supplementary Data 1). CRY32, CRY34, HY-029 and HY-037 are the most commonly shared races across geographic regions (Fig. S1). Of the sampled sites, Longnan (G4) had the highest virulence diversity, followed by Western Liupan Mountain (G3) (Table S6).

### KASP markers for *Pst* genotyping

A total of 1,790,286 variants, including SNPs and Indels, were identified after alignment. In the initial filtering, 1,381,576 bi-allelic SNPs with a depth range of 20 to 1000 were selected. From these, 100,749 independent SNPs were chosen with linkage disequilibrium below the threshold. Among them, 1076 were identified as homozygous sites across the 28 *Pst* isolates. A further rigorous filtering process based on minor allele frequency, missing rates, and other parameters resulted in 256 high-quality SNPs. In the final round of filtering, SNP sites adjacent to simple repeats and other SNPs were excluded, yielding 113 SNPs (Table S7). Fifty-seven SNPs were found to be suitable for designing allele-specific primers using Primer3 software. These 57 SNPs, distributed across all 18 chromosomes except for chromosome 15, were converted into KASP-SNP markers for genotyping. The minor allele frequency of these markers ranges from 0.11 to 0.39, with an average of 0.17. Gene diversity ranged from 0.19 to 0.48 with an average of 0.27. Polymorphism information content (PIC) varied from 0.17 to 0.36, with an average of 0.23. The heterozygous frequency was 0 for all the 57 SNPs (Table S8).

### Genetic diversity and structure of *Pst* population

We randomly selected 36 *Pst* isolates from the six geographic subpopulations to perform the initial genotypic characterization with 57 KASP-SNP markers, and selected 37 markers that led to clear genotypic clusters to evaluate *Pst* population structure for the six geographic groups. The genotype accumulation curve indicates that 37 loci are sufficient for discriminating individuals (Fig. S2). An average PIC value of 0.34 and gene diversity of 0.44 were observed when testing the markers in a population of 308 *Pst* isolates (Table S9). The overall population showed high genetic diversity with the Shannon’s information ranging from 0.59 to 0.63. Longnan (G4) group had the highest genetic diversity. Across all six geographic populations, the observed heterozygosity was lower than expected, except for the subpopulation in Middle Gansu (Table 1).

**Table 1:** The genetic diversity and heterozygosity of *Puccinia striiformis* f. sp. *tritici* in the sampled six geographic regions

Geographic regions	Number of isolates	*N*_*a*_	*N*_*e*_	*I*	*H*_*o*_	*H*_*e*_	*NP*
G1	119	2	1.79	0.62	0.40	0.44	37.00
G2	22	1.97	1.75	0.59	0.42	0.41	36.00
G3	61	2	1.79	0.62	0.39	0.43	37.00
G4	41	2	1.80	0.63	0.41	0.44	37.00
G5	42	2	1.78	0.62	0.37	0.43	37.00
G6	23	2	1.76	0.61	0.40	0.42	37.00
Total/Average	308	1.995	1.78	0.61	0.40	0.43	36.83

*N*_*a*_ number of observed alleles, *N*_*e*_ effective number of alleles, *I* Shannon’s information index, *H*_*o*_ observed heterozygosity, *H*_*e*_ expected heterozygosity, and *NP* number of polymorphic loci. G1 Eastern Qinghai, G2 Middle Gansu, G3 Western Liupan Mountain, G4 Longnan, G5 Eastern Liupan Mountain, and G6 Western Guanzhong Plain.

**Fig. 2:**
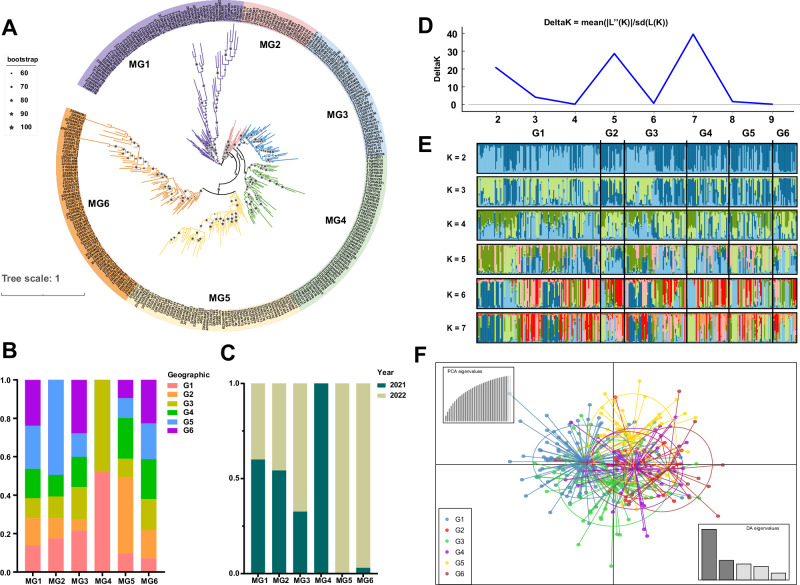
Population genetic structure of 308 *Puccinia striiformis* f. sp. *tritici* (*Pst*) isolates. **A** Phylogenetic tree based on 37 SNP markers. MG 1 to MG 6 with different background colors indicate molecular groups 1 to 6. Asterisks on the branches show bootstrap values. **B** Stacked column chart showing the composition of *Pst* isolates from geographic regions in each molecular group. **C** Stacked column chart showing the composition of *Pst* isolates sampled in 2021 and 2022. **D** Determination of the number of clusters using the statistic proposed by Evanno et al.^79^. **E** Ancestry coefficient analysis of the sampled *Pst* isolates with K (number of clusters) ranging from 2 to 7. **F** Population structure of sampled *Pst* isolates based on the discriminant analysis of principal components.

The 308 *Pst* isolates were clustered into six distinct groups, named as molecular group 1 (MG 1) to MG 6 (Fig. 2a), as confirmed by the STRUCRTURE analysis (Fig. S3). Most of the isolates from Eastern Qinghai (G1) and Western Liupan Mountain (G3) were grouped in MG4, accounting for 52.0% and 48.0%, respectively. *Pst* isolates from Middle Gansu (G2) were primarily (39.61%) in MG 5. Nearly 50% of the isolates from Eastern Liupan Mountain (G5) were in MG 2. Isolates from Longnan (G4) were evenly distributed across all MGs, except for MG4 which did not contain any isolates from Longnan. Isolates from Western Guanzhong Plain (G6) were also evenly distributed across MGs but without representation in MG 4 (Fig. 2b). Isolates sampled in 2022 were predominantly in MG 5 (100%) and MG 6 (96.97%), whereas MG 4 only contained isolates collected in 2021 (Fig. 2c).

Uneven distributions of MGs were observed across the geographic populations. MG4 was primarily found in G1 (32.77%) and G3 (29.51%), while other MGs showed relatively balanced distributions in these regions. In G2, MG5 was the predominant group, comprising 50.00% of the population, whereas no presence of MG4. Likewise, MG4 was absent in G4 and G5, where MG6 (31.71%) and MG1 (28.57%) were the most abundant groups, respectively. In G6, neither MG2 nor MG4 was detected, with MG6 (34.78%) being the predominant group in this region (Fig. S4). Further phylogenetic analysis of *Pst* isolates from Eastern Qinghai (G1), Middle Gansu (G2), and Eastern Liupan Mountain (G5) revealed admixture of isolates from the three geographic regions in Clusters 1, 2, 4, and 5. In Cluster 3, two isolates from G3 clustered with isolates from G1 (Fig. S5).

The additional investigations of *Pst* geographic populations were performed using STRUCTURE analysis. At K = 4, isolates from Eastern Qinghai (G1) formed a distinct group compared to other locations. Longnan (G4) and Eastern Liupan Mountain (G5) subpopulations appeared to be an admixture, sharing a similar genetic structure with the Western Guanzhong Plain (G6) subpopulation. Despite its geographical closeness to Eastern Qinghai (G1), the *Pst* subpopulation in Middle Gansu (G2) was similar to that of Longnan (G4) and Eastern Liupan Mountain (G5). The *Pst* subpopulation from Western Liupan Mountain (G3) appeared to be admixture of subpopulations from Eastern Qinghai (G1) and Longnan (G4)/Eastern Liupan Mountain (G5) (Fig. 2e).

DAPC analysis confirmed STRUCTURE results: distinct groups were observed for Eastern Qinghai (G1) and Western Guanzhong Plain (G6), whilst there were overlaps between Longnan (G4) and other locations, including Middle Gansu (G2), and Eastern (G5) and Western (G3) Liupan Mountain. Overlaps were also identified between subpopulations from Eastern Qinghai (G1) and Western Liupan Mountain (G3) (Fig. 2f). AMOVA showed that genetic variation was primarily within the geographic groups (accounting 97.84% of the total variation) (Table S10).

### Genetic exchanges

**Fig. 3:**
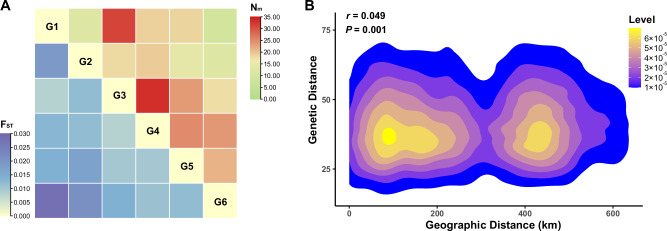
Genetic differentiation and isolation by distance analyses of *Puccinia striiformis* f. sp. *tritici* in the six geographical regions. **A** Pairwise genetic differentiation (*F*_*ST*_) and number of estimated migrants (*N*_*m*_) among the six *Pst* geographic regions. **B** Correlation analysis between geographic and genetic distances. The X-axis represents the geographic distance between pairs of the 308 isolates, while the Y-axis shows the genetic distance between pairs. The Level reflects the density of points representing the relationship between genetic and geographic distances. Areas with colors closer to yellow indicate a higher density of points within that range, while areas with a blue hue represent a lower density of points.

Substantial *Pst* genetic exchange between Eastern Qinghai (G1) and with Western Liupan Mountain (G3) was detected: *F*_*ST*_ = 0.008. In contrast, less exchange was detected between Eastern Qinghai (G1) and Western Guanzhong Plain (G6) (*F*_*ST*_ = 0.028). Middle Gansu (G2) had more genetic exchanges with Western Liupan Mountain (G3) (*F*_*ST*_ = 0.012) and Longnan (G4) (*F*_*ST*_ = 0.012), and less exchange with Western Guanzhong Plain (*F*_*ST*_ = 0.021). Western Liupan Mountain (G3) had considerable genetic exchanges with Longnan (G4) (*F*_*ST*_ = 0.008) as well as Eastern Qinghai (G1). Longnan (G4) also showed some genetic relationship with Eastern Liupan Mountain (G5) (*F*_*ST*_ = 0.010) and Western Guanzhong Plain (G6) (*F*_*ST*_ = 0.011). Despite being geographically separated by Liupan Mountain, *Pst* subpopulations in Eastern (G5) and Western (G3) areas had considerable amount of genetic exchange (*F*_*ST*_ = 0.010). As the recipient region from the oversummering region, the subpopulation in Western Guanzhong Plain (G6) predominantly originated from Longnan (G4) and Eastern Liupan Mountain (G5) with similar *F*_*ST*_ values (0.011 and 0.012) (Fig. 3a; Table S11). Estimated *N*_*m*_ values were consistent with the conclusions drawn based on *F*_*ST*_ analysis. Genetic exchanges rarely occurred between Eastern Qinghai (G1) and Western Guanzhong Plain (G6) (*N*_*m*_ < 10). In contrast, genetic exchanges were frequent between Eastern Qinghai (G1) and Western Liupan Mountain (G3), as well as between Longnan (G4) and Western Liupan Mountain (G3), with *N*_*m*_ > 30 (Fig. 3a; Table S11). For the remaining regions, moderate genetic exchanges were observed, with *N*_*m*_ values ranging between 11.510 and 25.003. The Mantel test indicated very low correlation between geographic and genetic distance (*r* = 0.05), albeit statistically significant (*P* = 0.01) (Fig. 3b). No significant association was detected between virulence and genotype in the *Pst* population (*r* = 0.024, *P* = 0.265) (Fig. S6).

### Air trajectory analysis of *Pst* in the oversummering regions

To determine potential dispersal routes of *Pst* among specific locations in oversummering region, we conducted air trajectory analyses. Air trajectories were simulated for the autumn (Oct. 1^st^ to Nov. 30^th^) and spring (Mar. 1^st^ to Apr. 30^th^), corresponding to *Pst* seedling infection in the autumn and the spring, respectively (Supplementary Data 2).

**Fig. 4:**
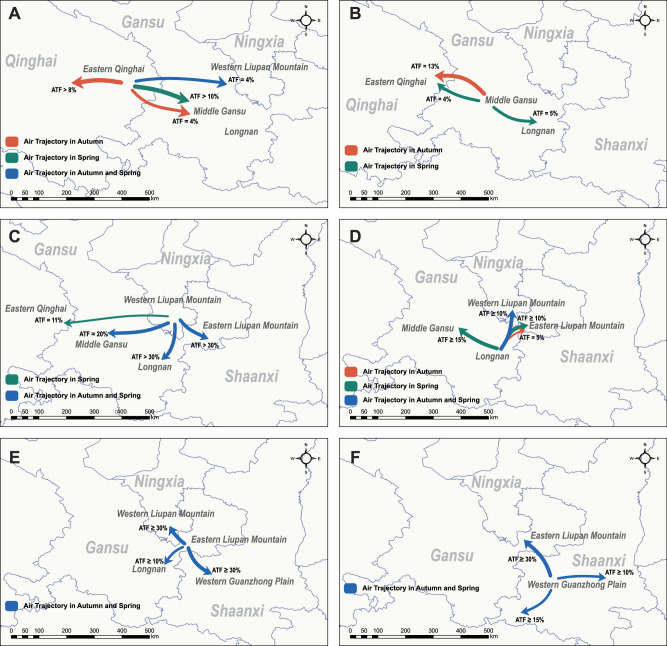
Simulated air trajectories for inferring *Puccinia striiformis* f. sp. *tritici*dispersal routes among geographic regions in the autumn and spring. The orange, green, and blue arrows denote simulated air trajectories in the autumn, and spring, and in both seasons, respectively. **A**–**F** The forward air trajectories started from Eastern Qinghai, Middle Gansu, Western Liupan Mountain, Longnan, Eastern Liupan Mountain, and Western Guanzhong Plain.

In Eastern Qinghai, air trajectories were primarily westward in autumn with an average trajectory frequency (ATF) of > 8% although wheat is rarely grown at this time in the region. *Pst* could also be spread to Western Liupan Mountain and Middle Gansu (ATF = 4%) within the same autumn (Fig. 4a, Fig. S7). In the spring, *Pst* inocula derived from Eastern Qinghai were mainly spread eastward to Middle Gansu (ATF > 10%) and Western Liupan Mountain (4%) (Fig. 4a, Fig. S7). In Middle Gansu, *Pst* were likely to disperse to Eastern Qinghai in the autumn (ATF = 13%) and spring (ATF = 4%), and to Longnan in the spring (ATF = 5%) (Fig. 4b, Fig. S8). Simulated air trajectories in Western Liupan Mountain indicted predominantly dispersal to Eastern Liupan Mountain and Longnan (with ATF > 30%), and Middle Gansu (ATF ~ 20%) in both autumn and spring. In addition, simulated air trajectories suggested that *Pst* could be spread to Eastern Qinghai in the spring (ATF = 11%) (Fig. 4c, Fig. S9). For all other locations, simulations suggested frequent dispersal routes between Longnan and Western Liupan Mountain (ATF ≥ 10%), between Western and Eastern Liupan Mountain (ATF ≥ 30%), between Longnan and Eastern Liupan Mountain (ATF ≥10%), and between Eastern Liupan Mountain and Western Guanzhong Plain (ATF ≥ 30%) in both the autumn and spring. Additionally, substantial *Pst* dispersal was possible between Longnan and Middle Gansu in the spring (ATF ≥ 15%) (Fig. 4d–f, Fig. S10–S12). In addition to Eastern Liupan Mountain, *Pst* inocula in Western Guanzhong Plain could be spread further eastwards to Central and Eastern Guanzhong Plain (ATF ≥ 10%) and southwards to Hanzhong Plain (ATF ≥ 15%) in both autumn and spring (Fig. 4f, Fig. S12).

### Field investigations

The onset of *Pst* at a number of sites in the oversummering region was monitored in 2021 and 2022. In Eastern Qinghai (G1), *Pst* was observed throughout the summer and autumn. From August to September in both 2021 and 2022, *Pst* was observed in the spring wheat during August and in both spring wheat and volunteer seedlings in September. In Middle Gansu (G2), *Pst* was first seen on volunteer seedlings in early September in both years. *Pst* occurrences in Western Liupan Mountain (G3), Longnan (G4), and Eastern Liupan Mountain (G5) followed a similar pattern, albeit a bit delayed (by two months in 2021, and one month in 2022), as the Eastern Qinghai (G1) and middle Gansu (G2). *Pst* was not observed in Western Guanzhong Plain during the autumn in both 2021 and 2022 but only seen in the subsequent spring (Table S12).

### Inference about *Pst* dispersal routes in the primary oversummering region

**Fig. 5:**
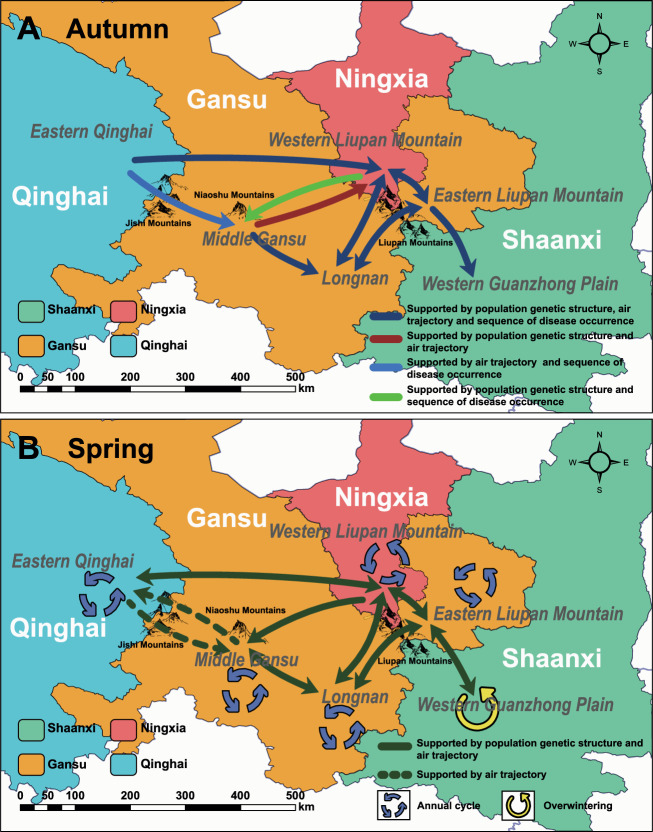
Inferred main dispersal routes of *Puccinia striiformis* f. sp. *tritici* from the northwestern oversummering region. Different arrows represent dispersal routes supported by specific evidence. **A** and **B** are the *Pst* dispersal routes in the autumn and spring, respectively.

Important *Pst* dispersal routes within the primary oversummering region were inferred based on spatial *Pst* genetic structure, simulated airflow trajectories, and observed temporal sequences of *Pst* occurrence at different locations. In autumn, oversummering *Pst* inocula on late-maturing spring wheat and/or volunteer seedlings in Eastern Qinghai are mainly dispersed to Western Liupan Mountain, initiating bidirectional exchanges of inoculum among the Western Liupan Mountain, Longnan, and Eastern Liupan Mountain regions. The *Pst* population of Longnan originates most likely from the oversummering inocula on volunteer seedlings in Middle Gansu and local oversummering inocula. In Western Guanzhong Plain, where *Pst* cannot oversummer, *Pst* inocula infecting autumn seedlings primarily originate from Eastern Liupan Mountain. Migration of *Pst* from Eastern Qinghai to Middle Gansu and bidirectional exchanges between Middle Gansu and Western Liupan Mountain are also possible (Fig. 5a). Additionally, more dispersal routes of *Pst* inocula are possible in spring. Consistent with the migration pattern in autumn, bidirectional migration of *Pst* inocula among Western Liupan Mountain, Longnan, and Eastern Liupan Mountain can occur continuously. Bidirectional *Pst* dispersal is likely to occur between Eastern Qinghai and Western Liupan Mountain, between Middle Gansu and Longnan, and between Eastern Liupan Mountain and Western Guanzhong Plain. A uni-directional dispersal may occur from Western Liupan Mountain to Middle Gansu. Potential *Pst* exchanges between Eastern Qinghai and Middle Gansu is supported by air trajectory analysis (Fig. 5b).

## Discussion

*Pst* dispersal varies among different territories because of differences in geographical characteristics and prevailing wind directions^17,36,37^. Changes in climate and cropping system in the *Pst* oversummering region in China are expected to affect the relative importance of *Pst* dispersal routes in the region. Among all the potential oversummering regions for *Pst* in China, the northwest, including Qinghai, Gansu and Ningxia, is the largest and most important region in terms of its contribution of *Pst* inocula to the main Chinese wheat production in spring^15,18,21,31^.

Over the past decade, previous investigations have examined the population structure and relationships of *Pst* in northwestern China. Among this region, extensive research has focused on Longnan, which is regarded as an important *Pst* inoculum source^15,31–35^. The dispersal routes of *Pst* have been confirmed from Longnan to Eastern Liupan Mountain and then to the Guanzhong Plain^15,35^. Additionally, inoculum from Longnan can spread to the Sichuan Basin and southern Shaanxi^15,31,33^, with frequent inoculum exchanges also inferred between Longnan and Western Liupan Mountain^32^. With changes in wheat cultivation practices, the importance of Eastern Qinghai has been highlighted due to the expansion of its *Pst* oversummering areas. Previous studies have primarily identified potential population exchanges between Longnan and Eastern Qinghai^15^, as well as a closer genetic relationship between Gansu (mainly central Gansu and Longnan) and Eastern Qinghai, compared to between Gansu and Xinjiang^34^. However, the overall dispersal routes of *Pst* in the northwestern oversummering regions are lacking, particularly between Eastern Qinghai and Longnan, as well as among Eastern Qinghai and other oversummering regions. The findings of the present study provide new insights into potential *Pst* dispersal routes and their relative significance in this region.

In recent years, changes in wheat production in the northwest oversummering region in China have led to an increased significance of Eastern Qinghai in terms of *Pst* oversummering and subsequent dispersal of *Pst* inocula to other wheat production areas. The present study demonstrated that *Pst* is present throughout the wheat growing season in Eastern Qinghai, particularly in August and September (key times for *Pst* oversummering). The increase in winter wheat production has further increased the significance of Eastern Qinghai in terms of *Pst* epidemic development in China. For instance, *Pst* inocula in Eastern Qinghai on late-maturing spring wheat and volunteer seedlings in August and September can be dispersed to Western Guanzhong Plain through Western and Eastern Liupan Mountain regions, as indicated by the close relationship between *Pst* subpopulations from the two regions. Additionally, *Pst* inocula from volunteer seedlings in Middle Gansu can be dispersed to Western Guanzhong Plain primarily through Longnan and Eastern Liupan Mountain and cause rust on autumn seedlings. *Pst* inocula in Middle Gansu is likely to originate from the east, according to simulated air trajectories and the temporal sequence of disease occurrence. However, the population structure suggested a low level of genetic exchanges between the two regions, which could be due to the separation by Jishi Mountain with the highest altitude of 4636 m. Once reached Guanzhong Plain, *Pst* inocula can cause rust autumn seedlings in the region and produce new inocula that can be further dispersed to Huang-Huai-Hai plain (the main wheat production area in China) in the late autumn^15,35^. Volunteer wheat plants act as a green bridge and/or the source of *Pst* on autumn seedlings in the oversummering region. The importance of volunteer crops in epidemic development has been demonstrated in other diseases, such as wheat leaf rust^38^, barley yellow dwarf^39^, and bacterial spot of tomato^40^. Eliminating volunteer crops is one of the effective management strategies to minimize oversummering inocula. In spring, despite the dispersal routes of *Pst* identified in this study, local overwintering inocula were also responsible for the spring epidemics. This was particularly evident in the regions of Eastern Qinghai and Middle Gansu, where the disease consistently appeared on the lower and older leaves during spring according to our long-term field monitoring, suggesting that the primary source of inocula in these regions is local rather than external. For other oversummering regions, more detailed investigations are needed to determine the sources of spring epidemics.

In the northwestern oversummering region, the lack of correlation between geographic and genetic distance among *Pst* subpopulations suggests that mountainous barriers in the region failed to stop long-distance *Pst* air dispersal. The overall low paired genetic differentiation and high number of migrants further illustrated that genetic exchange among *Pst* populations in this region is substantial, particularly between Western and Eastern Liupan Mountains and Longnan. This finding is consistent with the previously identified dispersal routes between Longnan and Eastern Liupan Mountain^15^, between Longnan and Western Liupan Mountain^31^, and between Eastern Liupan Mountain and Guanzhong Plain^35^. The Liupan Mountain divides the local wheat production areas into Western and Eastern subareas. The limited genetic exchange between the eastern and western sides of Liupan Mountain is believed to be due to the physical mountain barrier^32^. Although the two *Pst* subpopulations are supported by the present results based on phylogeny and population structure analysis, the present research also suggests substantial genetic exchanges between them.

Among the molecular groups identified through phylogeny, MG4 consists exclusively of isolates from Eastern Qinghai and Eastern Liupan Mountain, indicating frequent genetic exchange between these populations. This is further corroborated by the lowest *F*_*ST*_ value and the highest *N*_*m*_. Eastern Qinghai and Western Liupan Mountain were widely represented across all molecular groups. Our field disease surveys indicated that the *Pst* oversummering areas and the amount of inoculum in Eastern Qinghai are considerably larger than in other regions. This substantial inoculum could influence neighboring areas, leading to close genetic relationships between the *Pst* populations in Eastern Qinghai and those in other geographic regions. Western Liupan Mountain, located at the intersection of oversummering regions, was found to enable direct *Pst* exchange with all geographic regions except the Western Guanzhong region, contributing to its wide representation in molecular groups. Despite the overall high diversity of *Pst* geographic regions in the MGs, we found that the Western Guanzhong Plain population is the most narrowly distributed among all the geographic groups in the MGs. Other *Pst* geographic populations are found in at least five MGs, while Western Guanzhong Plain is represented in fewer groups, with no distribution in MG2 and MG4. The possible reason could be due to the topography; despite the relatively short geographic distance between Western Guanzhong Plain and Longnan, the Long Mountain acts as a barrier to the exchange of inoculum. Additionally, the Liupan Mountain also restricts the exchange of inoculum between Western Guanzhong Plain and Western Liupan Mountain. The geographic distance between Western Guanzhong Plain and Eastern Qinghai and Middle Gansu is considerable, with mountain ranges in between, limiting the exchange of inoculum with these regions. Another contributing factor is that the Guanzhong Plain is unsuitable for oversummering, making it a recipient of inoculum rather than a source, while bidirectional exchanges are more frequent in other oversummering regions, resulting in less frequent inoculum exchange in Western Guanzhong Plain. Among all the studied geographic populations, *Pst* in Longnan and Western Liupan Mountain had the greatest genetic diversity, including virulence, consistent with previous studies^15,20,30,31^. The dispersal routes inferred in the present study suggest that these two regions are central to the *Pst* inoculum movement with other neighboring wheat production areas. The high level of observed heterozygosity relative to the expected implies that the Middle Gansu region has been experiencing a high degree of *Pst* exchanges with its neighboring areas. The higher-than-expected heterozygosity, in contrast to the other regions, may also suggest the existence of possible selection favoring specific races in this area.

CYR32 is previously identified as the predominant race in 11 provinces of China, accounting for 34.6% of isolates sampled in 2001-2002^20^, and in 2003-2007^30^. Recent investigations indicated that CYR34 and CYR32 are the most prevalent races in China, with an increased prevalence of CYR34 in recent years^41,42^. Zhou et al.^42^ identified CYR34 as the predominant race across 16 provinces in China. However, Zhang et al.^41^ reported that CYR32 was the predominant race in northwest China in 2021, with both CYR32 and CYR34 dominating in 2022. Similarly, Yang et al.^43^ found CYR32 to be the predominant race in southwest China from 2020 to 2021. In the present study, CYR34 is the predominant race in Longnan, while CYR32 dominates in other regions. The discrepancies in predominant races across regions may result from differences in the locations and years from which the *Pst* isolates were sampled. By comparing the shared races across geographic regions, Eastern Qinghai harbors the highest number of shared races with other regions, aligning with the distribution of MGs, and suggesting its influence as an inoculum source. Regarding to exclusive races, Eastern Qinghai also obtains the highest number, reflecting a high diversity in race composition. In contrast, Western Guanzhong Plain shows the lowest number and proportion of exclusive races, conforming to limited exchanges with other regions. CRY32 is presented across all region pairs, making it the most prevalent race and a critical focus for wheat breeding programs. The emerging race CYR34, predominant in Longnan, is shared with Eastern Qinghai, Eastern Liupan Mountain, and Western Guanzhong Plain. Given the high races diversity in Eastern Qinghai, the presence of shared CYR34 is unsurprising. Shared CYR34 among Longnan, Eastern Liupan Mountain, and Western Guanzhong Plain indicate that inoculum from Longnan can disperse to Eastern Liupan Mountain and, under conditions of high inoculum density, directly to the Western Guanzhong Plain, consistent with previous findings^35^. Zhou et al.^42^ identified 119 races in 608 *Pst* isolates from 16 provinces across China, including 94 new races. The present study obtained 99 new races from 308 isolates, suggesting that the northwest oversummering region is a hotspot for the emergence of new races.

Mutation, somatic recombination, and sexual recombination have been proposed or confirmed as the primary genetic mechanisms driving virulence changes in *Pst*^9,13,44^. The sampling sites in our study are mainly located in the mountainous regions, where alternate hosts of *Pst* (*Berberis* species) are widely distributed^45,46^. The potential for sexual recombination in these regions could contribute to the high diversity of the race composition. Mutation is another factor influencing the virulence variation of *Pst*. Previous studies have shown that mutants with various virulence patterns can be induced by ultraviolet radiation^47,48^. Given that the sampling sites in *Pst* oversummering regions are primarily situated at high altitudes, with an average altitude of 2200.8 m, the strong ultraviolet radiation in these areas could accelerate the rate of point mutations, resulting in increased virulence variation and a higher number of *Pst* races^47–49^. Additionally, host selection may also play a role in shaping *Pst* race composition. The mountainous regions in the *Pst* oversummering areas are unsuitable for large-scale wheat cultivation. Instead, the smallholder farming system promotes a wide variety of wheat cultivars, further contributing to the high number of *Pst* races.

Our study also indicates that several *Pst* resistance genes - *Yr9*, *Yr1*, *YrSu*, *Yr3*, *YrJu1*, *YrJu2*, *YrJu3*, *YrVir1*, and *YrVr2* - are ineffective, whilst *Yr5* is still effective. Only one of 308 isolates was virulent to WD15 and WD18. WD15 carries an unknown resistance gene (possibly derived from *Thinopyrum intermedium*) whereas WD18 has *Yr5* from *Triticum spelta*. This highlights the importance of exploring *Pst* resistance genes from wheat-related species.

In population genetic studies of *Pst*, simple sequence repeats (SSRs) markers have been extensively used to explore genetic exchanges among populations in different regions worldwide^14,50–52^. Compared to SSRs, SNPs are the most abundant, widely distributed, and more stable genetic variation in genomes^53^. The advantages of SNPs, combined with KASP technology, provide a convenient and efficient approach for population genetic studies. In recent years, a few *Pst* population genetic studies have utilized KASP-SNPs developed by Meng et al. ^54^, who identified 43 SNPs by comparing raw reads from two *Pst* isolates originating in the US and India. In our study, we took a pioneering approach by developing a set of 37 homozygous KASP-SNPs from the genome of 28 global *Pst* isolates, offering a broader range of applications and enhanced polymorphism. Given the dikaryotic nucleus structure of *Pst*, this set of markers effectively avoids genotype inaccuracies caused by allele heterozygosity. We found no significant correlation between virulence and genotype. One possible explanation is that they evolve independently under different selective pressures. Virulence may evolve rapidly in response to host selection, particularly in regions with diverse wheat variety cultivation, while genotype is primarily shaped by environmental factors. Additionally, due to the lack of identified avirulence genes in *Pst*, another hypothesis is that the molecular markers we used may not be closely linked to the avirulence/virulence loci, making it difficult to detect a direct association with virulence.

In the present study, we performed spatial and temporal analysis among *Pst* subpopulations in the northwest *Pst* oversummering region in China to identify migration patterns in the region. Based on the results, we proposed two key *Pst* dispersal routes, which leads us to conclude that East Qinghai is of great importance as a source of *Pst* in China. Further research is needed to develop, implement, and evaluate *Pst* management strategies taking into account the importance of Eastern Qinghai.

## Method

### Sampling, multiplying, and identifying field *Pst* isolates

A total of 308 *Pst* samples were collected from 122 sites in the main *Pst* areas in the northwest oversummering region of China in 2021 and 2022. Sampled leaves with rust symptoms were placed on a piece of water-soaked paper towels within Petri dishes and kept in the dark at 10°C for 24 h before inoculation. To purify *Pst* inoculum, a single uredium was picked with a toothpick and transferred to seedlings of cv. Mingxian 169, a wheat cultivar susceptible to all known *Pst* races in China. The inoculated seedlings were maintained in a dew chamber at 10 °C for 24 h, then moved to greenhouse benches with temperatures ranging from 12 °C to 16 °C. To prevent cross-contamination, the seedlings were placed in glass booths. Urediniospores were harvested about 16 days post-inoculation. Subsequent steps, including inoculum multiplication, spore collection and storage, followed standard protocols^17,55^.

To assess avirulence/virulence of sampled *Pst* isolates, a set of 19 standard Chinese wheat differentials (WDs) together with cv. Mingxian 169 was used (Table S2). Around 10 days after planting, two-leaf stage seedlings were inoculated with freshly harvested urediniospores using the standard multiplication procedure^17,55^. Infection types (ITs) were recorded 18 to 20 days post-inoculation using a scale of 0 to 9^36^, once the susceptible cv. Mingxian 169 was fully diseased. ITs in the range of 0-6 were classified as avirulent, whilst those in the range of 7–9 as virulent. *Pst* races were determined according to the criteria proposed by CNWRCG^20,30^. Virulence Analysis Tool (VAT) (https://en-lifesci.tau.ac.il/profile/kosman/vat) was used to obtain *Pst* race frequencies at each geographic site.

### Pathotypic analysis based on the *Pst* virulence spectrum

VAT was used to calculate virulence diversity indices (Nei’s diversity index, Kosman index, Simpson index, Shannon and Weaver index, and Stoddart index) and to determine the pathotype structure among geographic regions based on Nei’s genetic distance^56^.

### Development of KASP markers of *Pst* isolates

Raw reads from 16 *Pst* isolates originating from the USA^57,58^, Australia^59,60^, United Kingdom^61^, Denmark^62^, France^61^, India^63^, and China^15^ were selected to represent the global *Pst* population (Table S13). Furthermore, additional 12 isolates from several *Pst* epidemic regions in China were sequenced (Table S14). These 28 error-corrected sets of raw reads were aligned to the reference genome CYR34 using BWA-MEM 0.7.17 followed by our previous settings^15^. Subsequently two rounds of variant calling were conducted using HaplotypeCaller in the Genome Analysis Toolkit (GATK) 4.3, as described previously^15,64^. The resulting genome variant call data were combined and genotyped using CombineGVCF and GenotypeGVCF in GATK.

To eliminate variants of low quality and retain only bi-allelic single nucleotide polymorphisms (SNPs), the initial variant call format (VCF) file was filtered with VCFtools 0.1.16^65^ with the parameters “--remove-indels --min-meanDP 20 --max-meanDP 1000 --min-alleles 2 --max-alleles 2”. To ensure that selected SNPs are not linked, those with a squared correlation coefficient ≤ 0.2 were kept using PLINK 1.90b7^66^ with the parameter “--indep-pairwise 50 1 0.2”. To ensure that the polymorphisms were not due to heterozygosity, heterozygous sites among 28 isolates were removed using GATK VariantFiltration. Further filtering was carried out with VCFtools using the parameters “--maf 0.1 --minQ 100 --max-missing 0.9 --thin 1000”. Additionally, sites within the 100 bp flanking selected SNPs that contained simple repeats were excluded.

Primers were designed from the selected SNPs and their flanking sequences using the Primer3 software with default settings (https://primer3.org/). Summary statistics, including minor allele frequency, gene diversity, polymorphic information content, and heterozygosity, were calculated using the PowerMarker 3.25^67^. SNP markers were transformed into Kompetitive Allele-Specific PCR (KASP) markers through the addition of the prefix sequences 5’ GAAGGTGACCAAGTTCATGCT 3’ for the FAM tail and 5’ GAAGGTCGGAGTCAACGGATT 3’ for the HEX tail. These sequences were appended to the forward and reverse primers corresponding to the wild type and mutant, respectively, as derived from the Primer3 software. The primers designed were synthesized by Sangon Biotech Co., Ltd. (Shanghai, China).

### DNA extraction, genotyping, and genetic diversity

Genomic DNA extraction from urediniospores was carried out with an improved cetyltrimethylammonium bromide (CTAB) method^55,68^. The quality and quantity of genomic DNA were assessed with gel electrophoresis (0.8% agarose) and a NanoDrop 2000 spectrophotometer (Thermo Fisher Scientific, Waltham, MA, USA). *Pst* isolates were genotyped with a set of 37 KASP-SNP markers. In a 384-well plate, 2 μL of genomic DNA was added to each well and dried at 65 °C in an incubator. Each well then received 2.5 μL of KASP Mastermix, 0.07 μL of mixed primers, and 2.5 μL of ddH_2_O. PCR amplification was conducted in a thermal cycler (Applied Biosystems, CA, USA) under the following conditions: pre-denaturation at 94°C for 15 min; 10 touchdown cycles with denaturation at 94°C for 20 s, annealing for 60 s at which temperature initially (decreasing 0.8 °C per cycle); followed by 32 cycles with denaturation at 94 °C for 20 s, annealing at 57 °C for 60 s. PCR-amplified products were analyzed with the FLUOstar Omega SNP and Kluster Caller software (LGC Biosearch Technologies, UK). Genetic diversity was measured by several indices, including the observed number of alleles (*Na*), effective number of alleles (*Ne*), number of polymorphic loci, percentage of polymorphic loci (*P*), Shannon’s information index (*I*), and expected (*He*) and observed (*Ho*) heterozygosity. These indices were calculated from genotypic data for all isolates as well as for each individual site via GENALEX 6.501 program^69^.

### Population structure and phylogenetic analysis

A total of 37 SNPs were used to construct a phylogenetic tree of 308 isolates using the maximum likelihood method. A bootstrapping analysis (with 1000 replications) was performed using the TVM + F + ASC + G4 model via the IQ-TREE 2.1.3 program^70^. The phylogenetic tree was visualized and annotated with iTOL 6 (https://itol.embl.de/). Nodes were considered well-supported if they had a bootstrap value of at least 60%. The geographic population distribution of *Pst* isolates within each molecular group (MG) was calculated. To balance the differences among geographic groups, we used the sample size of the largest group to divide sample sizes of each group to determine weighting factors, which were applied to calculate the proportion of each MG. Population structure analysis was carried out with the STRUCTURE 2.3 program^71^. For each simulated cluster size (K), five runs were conducted with a burn-in period of 10,000 and 40,000 iterations. The optimal K value was determined with the STRUCTURE HARVESTER program (http://taylor0.biology.ucla.edu/structureHarvester). The resulting population structure was processed with the Clumpp 1.1.2 program^72^, and visualized in Distruct 1.1^73^. In addition, Discriminant Analysis of Principal Components (DAPC), as implemented in the *adegenet* R package^74^, was used to identify and describe clusters of sampled isolates. A hierarchical analysis of molecular variance (AMOVA) was used to evaluate the partition of genetic variance into among or within subpopulations defined by sample locations^75^.

### Population differentiation, genetic exchanges and the relationship between virulence and genotype

Genetic relationships among *Pst* subpopulations at different sites were assessed through analysis of the fixation index (*F*_*ST*_). The number of migrants (*N*_*m*_) was estimated with the formula *N*_*m*_ = 0.25 $\times$ (1/ *F*_*ST*_ $-$ 1)^76^. The correlations between geographic and genetic distances, as well as between virulent and genetic distances, were evaluated using the Mantel test the vegan v2.6-4 R package (https://cran.r-project.org/web/packages/vegan/index.html). Geographic distances between sampling sites were computed based on their latitude and longitude coordinates. Genetic and virulent distances were calculated using Nei’s genetic distance formula^77^.

### Field survey and air trajectory simulation

*Pst* field surveys were conducted in 2021 and 2022 to record the temporal sequence of disease onset across the northwestern oversummering region. Assessment included wheat growth stage, and *Pst* incidence and severity. Air trajectory simulations were performed with the Lagrangian Integrated Trajectory 4 (HYSPLIT-4) program based on weekly meteorological data in 2021 and 2022. Meteorological data were provided by the National Oceanic and Atmospheric Administration (NOAA) (ftp://arlftp.arlhq.noaa.gov/pub/archives/gdas1). The simulated period and altitude range were selected based on the time of the disease outbreak and the topographic features of various areas. The frequency of air trajectories (as a proxy for *Pst* dispersal) from each site at 100 to 3000 m AGL was calculated. The duration for each simulation was set to 120 h, as *Pst* spores may survive in the air for up to 120 h^78^. Average trajectory frequency (ATF) originating from each location was computed and visualized with *ggplot2*, *tmap*, and *sp* R packages.

### Statistics and reproducibility

To ensure the accuracy of virulence test results, five seeds from each wheat differential hosts were planted as replicates in each corner of the pots. A total of five pots were used for the virulence test of each isolate. For each isolate, the ITs of five replicated seedlings of each WD were evaluated individually. Due to the possibility of disease escape, the most frequent ITs observed in each differential line were recorded. Correlation analyses were performed to examine the relationships between geographic and genetic distances, and between virulence and genetic distances. All the statistical analyses were computed in mantel() function of vegan v2.6-4 R package, with 9999 permutations. A threshold of 0.05 for the *P* value was applied to determine statistical significance. All analyses can be reproduced using the data and code provided in the Data Availability and Code Availability statements.

### Reporting summary

Further information on research design is available in the Nature Portfolio Reporting Summary linked to this article.

## Supplementary information

Supplementary Information

Description of Additional Supplementary Files

Supplementary Data 1

Supplementary Data 2

Reporting Summary

## Data Availability

Whole-genome sequencing data of 12 *Pst* raw reads have been deposited at the National Center for Biotechnology Information in SRA accessions SRR27902522 to SRR27902533 under BioProject PRJNA1073700.

## Code availability

The code used in this study is described in the Methods section and is available on GitHub at https://github.com/yuxiang-li/Pst_oversummer_population.
