# Supplementary material for: Migration of wheat stripe rust from the primary oversummering region to neighboring regions in China
Source: Commun Biol. 2025 Mar 3;8:350. doi: 10.1038/s42003-025-07789-3 (PMC11876435; doi:10.1038/s42003-025-07789-3)
Supplement: Supplementary file 2 — Description of Additional Supplementary Files [file 42003_2025_7789_MOESM2_ESM.pdf]

Description of Additional Supplementary Files

File name: Supplementary Data 1

Description: Shared and individual races of the stripe rust pathogen in six geographic regions.

File name: Supplementary Data 2

Description: Raw data for trajectory analyses in six geographic regions during both Spring and Autumn.
