# Supplementary material for: Migration of wheat stripe rust from the primary oversummering region to neighboring regions in China
Source: Commun Biol. 2025 Mar 3;8:350. doi: 10.1038/s42003-025-07789-3 (PMC11876435; doi:10.1038/s42003-025-07789-3)
Supplement: Supplementary file 5 — Reporting Summary [file 42003_2025_7789_MOESM5_ESM.pdf]

nature portfolio

Corresponding author(s): Xiaoping Hu, Qiang Yao

Last updated by author(s): Feb 19, 2025

Reporting Summary

Nature Portfolio wishes to improve the reproducibility of the work that we publish. This form provides structure for consistency and transparency in reporting. For further information on Nature Portfolio policies, see our Editorial Policies and the Editorial Policy Checklist.

Statistics

For all statistical analyses, confirm that the following items are present in the figure legend, table legend, main text, or Methods section.

n/a	Confirmed	
☐	☒	The exact sample size (n) for each experimental group/condition, given as a discrete number and unit of measurement
☒	☐	A statement on whether measurements were taken from distinct samples or whether the same sample was measured repeatedly
☐	☒	The statistical test(s) used AND whether they are one- or two-sided<br>*Only common tests should be described solely by name; describe more complex techniques in the Methods section.*
☒	☐	A description of all covariates tested
☒	☐	A description of any assumptions or corrections, such as tests of normality and adjustment for multiple comparisons
☐	☒	A full description of the statistical parameters including central tendency (e.g. means) or other basic estimates (e.g. regression coefficient) AND variation (e.g. standard deviation) or associated estimates of uncertainty (e.g. confidence intervals)
☐	☒	For null hypothesis testing, the test statistic (e.g. F, t, r) with confidence intervals, effect sizes, degrees of freedom and P value noted<br>*Give P values as exact values whenever suitable.*
☐	☒	For Bayesian analysis, information on the choice of priors and Markov chain Monte Carlo settings
☒	☐	For hierarchical and complex designs, identification of the appropriate level for tests and full reporting of outcomes
☒	☐	Estimates of effect sizes (e.g. Cohen's d, Pearson's r), indicating how they were calculated

Our web collection on statistics for biologists contains articles on many of the points above.

Software and code

Policy information about availability of computer code

Data collection	No software was used for primary data collection. NOAA data were used as part of the data interpretation as detailed in the Methods.
Data analysis	Air trajectory simulations were carried out by HYSPLIT 4 using NOAA data. Meteorological data were provided by the National Oceanic and Atmospheric Administration (NOAA) (ftp://arlftp.arlhq.noaa.gov/pub/archives/gdas1/) .The simulated period and altitude range were selected based on the time of disease outbreak and the topographic features of various areas.. Average trajectory frequency (ATF) originating from each location was computed and visualized with ggplot2, tmap, and sp R packages. Whole-genome sequencing data of 16 Pst raw reads have been deposited in the National Center for Biotechnology Information under BioProject database PRJNA1073700. Additional Rpackages, vegan, adegenet were also used in plotting and analysis. All codes used in the analyses relevant to this paper are available as explained in the Code Availability.

For manuscripts utilizing custom algorithms or software that are central to the research but not yet described in published literature, software must be made available to editors and reviewers. We strongly encourage code deposition in a community repository (e.g. GitHub). See the Nature Portfolio guidelines for submitting code & software for further information.

Data

Policy information about availability of data

All manuscripts must include a data availability statement. This statement should provide the following information, where applicable:

- Accession codes, unique identifiers, or web links for publicly available datasets
- A description of any restrictions on data availability
- For clinical datasets or third party data, please ensure that the statement adheres to our policy

Whole-genome sequencing data of 12 Pst raw reads have been deposited at the National Center for Biotechnology Information in SRA accessions SRR27902522 to SRR27902533 under BioProject PRJNA1073700.

Human research participants

Policy information about studies involving human research participants and Sex and Gender in Research.

Reporting on sex and gender	Not applicable.
Population characteristics	Not applicable.
Recruitment	Not applicable.
Ethics oversight	Not applicable.

Note that full information on the approval of the study protocol must also be provided in the manuscript.

Field-specific reporting

Please select the one below that is the best fit for your research. If you are not sure, read the appropriate sections before making your selection.

☐ Life sciences ☐ Behavioural & social sciences ☒ Ecological, evolutionary & environmental sciences

For a reference copy of the document with all sections, see nature.com/documents/nr-reporting-summary-flat.pdf

Life sciences study design

All studies must disclose on these points even when the disclosure is negative.

Sample size	
Data exclusions	
Replication	
Randomization	
Blinding	

Behavioural & social sciences study design

All studies must disclose on these points even when the disclosure is negative.

Study description	
Research sample	
Sampling strategy	
Data collection	
Timing	

Data exclusions	
Non-participation	
Randomization	

Ecological, evolutionary & environmental sciences study design

All studies must disclose on these points even when the disclosure is negative.

Study description	we developed a set of KASP-SNP marker to investigate the population structure in the Puccinia striiformis f. sp. tritici oversummering and its adjacent regions. A set of 19 Chinese wheat differentials were used to characterize the virulence patterns of 308 sampled Pst isolates. By integrating virulence characterization, population genetic analysis, air trajectory simulation and field disease monitoring, two main Pst dispersal routes were identified.
Research sample	As we aim to unravel the Pst migration pattern originating from oversummering regions under changing agriculture practices, we collect 308 Pst samples were collected from 122 sites in the main Pst areas in the northwest oversummering region of China in 2021 and 2022.
Sampling strategy	Changing climate has led to significant changes in cropping systems in the Pst oversummering region. This pattern facilitates the spread of oversummering inocula from spring wheat to local winter wheat, significantly influencing Pst development in other areas. In response to the changes in climate and cropping systems in recent years, we collected samples mainly from the over-summering areas of Pst and the adjacent regions. For the field with disease center, we collected each sample from each disease center. For the field with widespread disease, we randomly collect 3 samples in the field.
Data collection	Whole-genome sequencing data of 16 Pst raw reads have been deposited in the National Center for Biotechnology Information under BioProject database PRJNA1073700. Meteorological data were provided by the National Oceanic and Atmospheric Administration (NOAA) (ftp://arlftp.arlhq.noaa.gov/pub/archives/gdas1/) .
Timing and spatial scale	The samples were collected the over-summering areas and the adjacent regions. The details of each sample are shown in the supplementary Table S1.
Data exclusions	The loss of samples during the amplification process is not included in the study.
Reproducibility	To ensure the reproducibility we tested the 37 loci using data from other Pst populations in China and demonstrated that the loci were reliable. Airflow trajectory analysis and field survey data were added to support the reliability of the results.
Randomization	Not applicable.
Blinding	Blinding was not possible during data collection because visible differences in plant phenotypes (e.g., disease symptoms and signs) made it easy to distinguish diseased plants.

Did the study involve field work? ☒ Yes ☐ No

Field work, collection and transport

Field conditions	Pst field surveys were conducted in 2021 and 2022 to record the temporal sequence of disease onset across the northwestern oversummering region. Assessment included wheat growth stage, and Pst incidence and severity.
Location	The location of wheat stripe rust field survey in different geographic regions in 2021 and 2022 are shown in the supplementary Table S12.
Access & import/export	Collected samples were transfered following existing procedures.
Disturbance	We believe that the 308 samples of Pst collected in the main overwintering areas identified in the survey are plausible as representative samples, given the spatial proportions of the actual sampling.

Reporting for specific materials, systems and methods

We require information from authors about some types of materials, experimental systems and methods used in many studies. Here, indicate whether each material, system or method listed is relevant to your study. If you are not sure if a list item applies to your research, read the appropriate section before selecting a response.

Materials & experimental systems

n/a	Involved in the study
☒	☐ Antibodies
☒	☐ Eukaryotic cell lines
☒	☐ Palaeontology and archaeology
☐	☒ Animals and other organisms
☒	☐ Clinical data
☒	☐ Dual use research of concern

Methods

n/a	Involved in the study
☒	☐ ChIP-seq
☒	☐ Flow cytometry
☒	☐ MRI-based neuroimaging

Antibodies

Antibodies used	
Validation	

Eukaryotic cell lines

Policy information about cell lines and Sex and Gender in Research

Cell line source(s)	
Authentication	
Mycoplasma contamination	
Commonly misidentified lines (See ICLAC register)	

Palaeontology and Archaeology

Specimen provenance	
Specimen deposition	
Dating methods	

☐ Tick this box to confirm that the raw and calibrated dates are available in the paper or in Supplementary Information.

Ethics oversight	

Note that full information on the approval of the study protocol must also be provided in the manuscript.

Animals and other research organisms

Policy information about studies involving animals; ARRIVE guidelines recommended for reporting animal research, and Sex and Gender in Research

Laboratory animals	The study did not involve laboratory animals.
Wild animals	Only Puccinia striiformis f. sp. tritici are included in this paper.
Reporting on sex	Not applicable.
Field-collected samples	Almost all samples collected were kept in field conditions for several days before they were moved to a freezer.
Ethics oversight	No ethical approval is required for sampling Puccinia striiformis f. sp. tritici.

Note that full information on the approval of the study protocol must also be provided in the manuscript.

Clinical data

Policy information about clinical studies

All manuscripts should comply with the ICMJE guidelines for publication of clinical research and a completed CONSORT checklist must be included with all submissions.

Clinical trial registration	
Study protocol	
Data collection	
Outcomes	

Dual use research of concern

Policy information about dual use research of concern

Hazards

Could the accidental, deliberate or reckless misuse of agents or technologies generated in the work, or the application of information presented in the manuscript, pose a threat to:

No	Yes	
☐	☐	Public health
☐	☐	National security
☐	☐	Crops and/or livestock
☐	☐	Ecosystems
☐	☐	Any other significant area

Experiments of concern

Does the work involve any of these experiments of concern:

No	Yes	
☐	☐	Demonstrate how to render a vaccine ineffective
☐	☐	Confer resistance to therapeutically useful antibiotics or antiviral agents
☐	☐	Enhance the virulence of a pathogen or render a nonpathogen virulent
☐	☐	Increase transmissibility of a pathogen
☐	☐	Alter the host range of a pathogen
☐	☐	Enable evasion of diagnostic/detection modalities
☐	☐	Enable the weaponization of a biological agent or toxin
☐	☐	Any other potentially harmful combination of experiments and agents

ChIP-seq

Data deposition

☐ Confirm that both raw and final processed data have been deposited in a public database such as GEO.

☐ Confirm that you have deposited or provided access to graph files (e.g. BED files) for the called peaks.

Data access links<br>*May remain private before publication.*	
Files in database submission	
Genome browser session<br>(e.g. UCSC)	

Methodology

Replicates	
Sequencing depth	

Antibodies

Peak calling parameters

Data quality

Software

Flow Cytometry

Plots

Confirm that:

- [ ] The axis labels state the marker and fluorochrome used (e.g. CD4-FITC).
- [ ] The axis scales are clearly visible. Include numbers along axes only for bottom left plot of group (a 'group' is an analysis of identical markers).
- [ ] All plots are contour plots with outliers or pseudocolor plots.
- [ ] A numerical value for number of cells or percentage (with statistics) is provided.

Methodology

Sample preparation

Instrument

Software

Cell population abundance

Gating strategy

- [ ] Tick this box to confirm that a figure exemplifying the gating strategy is provided in the Supplementary Information.

Magnetic resonance imaging

Experimental design

Design type

Design specifications

Behavioral performance measures

Acquisition

Imaging type(s)

Field strength

Sequence & imaging parameters

Area of acquisition

Diffusion MRI ☐ Used ☐ Not used

Preprocessing

Preprocessing software

Normalization

Normalization template

Noise and artifact removal

Volume censoring

Statistical modeling & inference

Model type and settings

Effect(s) tested

Specify type of analysis: ☐ Whole brain ☐ ROI-based ☐ Both

Statistic type for inference
(See Eklund et al. 2016)

Correction

Models & analysis

n/a	Involved in the study
☐	☐ Functional and/or effective connectivity
☐	☐ Graph analysis
☐	☐ Multivariate modeling or predictive analysis

Functional and/or effective connectivity

Graph analysis

Multivariate modeling and predictive analysis
